# Congenital cytomegalovirus infection: the state of the art and future perspectives

**DOI:** 10.3389/fped.2023.1276912

**Published:** 2023-11-16

**Authors:** S. Salomè, F. R. Corrado, L. L. Mazzarelli, G. M. Maruotti, L. Capasso, D. Blazquez-Gamero, F. Raimondi

**Affiliations:** ^1^ Division of Neonatology, Department of Translational Medical Sciences, University of Naples Federico II, Naples, Italy; ^2^ Division of Obstetrician and Gynecologist, Department of Translational Medical Sciences, University of Naples Federico II, Naples, Italy; ^3^ Pediatric Infectious Diseases Unit, Hospital Universitario 12 de Octubre, Instituto de Investigación Hospital 12 de Octubre (Imas12), Translational Research Network in Pediatric Infectious Diseases (RITIP), Universidad Complutense, Madrid, Spain

**Keywords:** congenital CMV, congenital cytomegalovirus (cCMV) infection, congenital cytomegalovirus, review, congenital cytomegalovirus disease

## Abstract

Congenital cytomegalovirus (cCMV) infection is the most common congenital infection, with an estimated incidence of approximately one in 200 infants in high-income settings. Approximately one in four children may experience life-long consequences, including sensorineural hearing loss and neurodisability. Knowledge regarding prevention, diagnosis, and treatment increased in the recent years, but some challenges remain. In this review, we tried to summarize the current knowledge on both the obstetrical and pediatric areas, while also highlighting controversial aspects and future perspectives. There is a need to enhance awareness among the general population and pregnant women through specific information programs. Further research is needed to better define the classification of individuals at birth and to have a deeper understanding of the long-term outcomes for so defined children. Finally, the availability of valaciclovir medication throughout pregnancy, where appropriate, has prompted the assessment of a universal serological antenatal screening. It is recommended to establish a dedicated unit for better evaluation and management of both mothers and children.

## 1. Introduction

Congenital cytomegalovirus (cCMV) infection is caused by *in utero* mother-to-fetus transmission and is a leading cause of birth defects and developmental disabilities. In fact, CMV is the most common cause of congenital infection (1), leading to life-long consequences, including sensorineural hearing loss (SNHL) and neurodisabilities (2).

Knowledge has been increased during the years, but there are still gray areas that necessitate further evaluation and research to enhance clinical management. These findings were subject to recent expert evaluation (3–5). However, this review builds upon this existing knowledge by incorporating novel evidence and proposing avenues for future investigation.

## 2. Pathogenesis

CMV is a member of the *Herpervirididae* family. Human CMV has a high degree of species specificity, with humans being the exclusive host for this virus. It is endemic worldwide and does not exhibit any seasonal variations. Transmission can occur by direct or indirect person-to-person contact via urine and oropharyngeal, cervical, and vaginal secretions; semen; milk; tears; blood products; or organ transplants. The virus exhibits a prolonged period of viral shedding, particularly after the primary infection (4).

The cell-mediated spread of the virus begins after a replication phase. The main host cells infected by CMV are the monocytes, macrophages, and endothelial cells, although CMV can replicate also in other cell types. The dissemination of the virus is hematogenous. The main secondary sites of host replication are the spleen and liver. Dissemination and replication are not completely controlled by host immunity, and CMV can remain latent mainly in the monocytes after the primary infection. Moreover, the viral genome is highly variable, and immunity against infection is incomplete. Therefore, in seropositive hosts, reinfection with different strains, as well as the reactivation of endogen latent strain, is possible. Those two types of infections can be classified as non-primary infections (NPIs), both of which are characterized by viral replication. These episodes typically manifest as asymptomatic in immunocompetent individuals, while they can result in severe disease in immunocompromised hosts (4).

## 3. Epidemiology

The seroprevalence of CMV infection increases with age and is higher in individuals with lower socioeconomic status, both in high- and low/middle-income countries. Seroprevalence among women of childbearing age varies also accordingly with those factors. The seroprevalence of CMV IgG among women of reproductive age in Europe as a whole, developed European countries, Japan, Latin America, and North America was found to be 45.6%–95.7%, 45.6%–65.9%, 60.2%, 58.3%–94.5%, and 24.6%–81.0%, respectively (6).The prevalence rate of primary infection in pregnancy (maternal primary infection, MPI) was approximately 1%–2% in Western Europe and in the United States, exhibiting sharp racial/ethnic disparities, specifically affecting pregnant women of non-Hispanic blacks and Mexican Americans, as well as their infants born with cCMV (7, 8). Being young and having at least one child are risk factors for MPI. Previous seronegative women conceiving within 2 years from their first pregnancy had a 19-fold and fivefold higher risk of primary fetal infection in the first trimester and of related sequelae in their infant, respectively, compared with the general population (9). Maternal NPI occurs when the pregnant woman has pre-existing CMV immunity but is exposed to a different strain (reinfection), or has a reactivation of a latent infection. The prevalence of NPI is not well defined and has been estimated at an annual rate of 10% among young women in the United States (10).

CMV is the most common cause of congenital infection globally, with an estimated pooled overall prevalence rate of cCMV of 0.67%, ranging from 0.48% in high-income countries to 1.42% in low/middle-income countries (11). Higher rates of cCMV are related to higher maternal CMV seroprevalence (and subsequent maternal NPI during pregnancy), higher population-level HIV prevalence, lower socioeconomic status, and younger mean maternal age (11).

## 4. Maternal infection

CMV is transmitted through direct contact with infected body fluids, and pregnant women most commonly acquire infection through exposure to the saliva and urine of young children, particularly their own children (12).

Maternal CMV infection in pregnancy can be primary or non-primary (reinfection or reactivation), and both can result in infection of the fetus, with similar consequences for the infant (13).

The risk of fetal transmission is approximately 30%–35% (14) after MPI and lower with NPI (1%–3%) (15).

The timing of maternal CMV infection in pregnancy influences the risk of vertical transmission and the severity of symptoms in the infant. In fact, vertical transmission after maternal primary CMV infection increases with gestational age; however, severe consequences are essentially limited to first trimester infection (see Table 1) (16).

**Table 1:** Vertical transmission rate and fetal insult after MPI (16).

	Vertical transmission rate	Fetal insult	Symptomatic at birth
Pre-conceptional	5.5% (95% CI: 0.1–10.8%)	NA	NA
Periconceptional	21% (95% CI: 8.4–33.6)	28.8% (95% CI: 2.4–55.1)	1.3% (95% CI: 0–4.5)
First trimester	36.8% (95% CI: 31.9–41.6)	19.3% (95% CI: 12.2–26.4)	9.1% (95% CI: 2.7–15.6)
Second trimester	40.3% (95% CI: 35.5–45.1)	0.9% (95% CI: 0–2.4)	0.3% (95% CI: 0–1.1)
Third trimester	66.2% (95% CI: 58.2–74.1)	0.4% (95% CI: 0–1.5)	0.4% (95% CI: 0–1.6)

NA, not available.

In high-income countries with a low seroprevalence, approximately half of the infected newborns are from maternal NPI (reactivation or reinfection) (17), while in countries with high seroprevalence, 90% of congenital infections are due to NPI (1). In countries with low to intermediate seroprevalence (as France), the risk to deliver an infected baby was four-fold higher in women who were seronegative before their pregnancy. In detail, the risk to deliver an infected baby after MPI was increased in younger women who have had almost a full-term pregnancy, born in high resources countries, and from higher-income groups. Conversely, the only two risk factors associated with delivering an infected baby after NPI include being young and unemployed (17).

Considering the higher risk of fetal transmission, MPI in the first trimester is the highest risk condition, although all women of childbearing age are at risk of contracting CMV and transmitting the virus to a fetus when pregnant.

## 5. Diagnosis of CMV infection in pregnancy

Maternal CMV infection in pregnancy, both primary and non-primary, is commonly asymptomatic. The symptoms are non-specific, such as mild fever, asthenia, myalgia, and flu-like syndrome, and observed only in one-third of the cases. Non-specific laboratory findings are detected in a half of the cases, mainly as lymphocytosis greater than 40% and elevated liver enzymes (18). For this reason, the diagnosis of CMV infection in pregnancy is challenging, particularly in the absence of routine antenatal screening.

Serological testing can only diagnose MPI, while it is often unhelpful in NPI. Seroconversion identifies primary infection. When it cannot be demonstrated, the diagnosis is based on a combination of IgG and IgM testing pattern.

Routine antenatal serological screening of pregnant women is not recommended in most countries, including Italy, but is applied at a local or regional level in some cases (19). In cases where antenatal screening is not recommended, testing is offered only to pregnant women presenting with suggestive clinical symptoms or signs during antenatal ultrasound scans (see Table 2). When performed, serology screening in pregnancy is based on IgG and IgM testing followed by IgG avidity testing in cases of positive IgM (23). Performing a second IgM test after 2 weeks to confirm the first result could delay the diagnosis and the subsequent therapy, so this strategy is not recommended. The presence of anti-CMV IgM in a pregnant woman's serum should not be solely relied upon as a definitive indicator of vertical transmission, as it lacks specificity for recent primary infections. It is crucial to note that IgM antibodies can persist for extended periods or may arise due to cross-reactivity with other viral infections. Therefore, additional diagnostic measures should be employed to confirm the presence of active CMV infection and to accurately assess it. Therefore, if positive IgM is detected, it is recommended to request IgG avidity testing to exclude or confirm a recent primary infection. In fact, a low avidity IgG implies a primary infection in the last 3 months, while a high avidity IgG implies an infection that occurred more than 3 months earlier. The avidity test may yield inconclusive results in approximately 0.5% of all women who undergo screening (4). In conclusion, this strategy has a good specificity to exclude primary infection, but its sensitivity in accurately diagnosing primary infection has not yet been evaluated. All efforts must be made to ascertain the timing of maternal infection, since it influences the risk of vertical transmission and the risk of long-term sequelae.

**Table 2:** Features reported to be associated with congenital CMV infection (20, 21).

Antenatal features	Postnatal features
**Maternal**	**Features found on clinical examination**
◦ Symptomatic CMV infection	◦ Small for gestational age (birth weight <-2 SD for GA)
◦ Cholestasis of pregnancy	◦ Microcephaly (head circumference <-2 SD for GA)
◦ Placental dysfunction	◦ (Prematurity^a^)
◦ CMV IgG seroconversion	◦ Petechiae, purpura (usually found within hours of birth and persist several weeks) or blueberry muffin rash (intra dermal hematopoiesis)
	◦ Jaundice (early and prolonged)
	◦ Hepatomegaly
	◦ Splenomegaly
	◦ Neurological signs with no other explanations (lethargy, hypotonia, seizures, poor sucking reflex)
**Fetal (Antenatal abnormalities on US or MRI):**	**Features found on laboratory evaluations**
◦ Cerebral abnormalities: ventriculomegaly (mild to moderate <15 mm or severe >20 mm), intracranial calcifications, microcephaly, subependymal cysts	◦ Anemia
	◦ Thrombocytopenia (occurs in the first week but platelets often increase spontaneously after the second week)
	◦ Leukopenia, isolated neutropenia
	◦ Elevated liver enzymes (ALT/AST)
◦ Extracerebral abnormalities: fetal growth restriction, hyperechogenic bowel, hepatomegaly, liver calcifications, pericardial effusion	◦ Conjugated hyperbilirubinemia
	◦ Abnormal cerebrospinal fluid indices, positive CMV DNA
* *	**Features found on neuroimaging**
	◦ Calcifications (often periventricular), ventricular dilatation without other explanations, periventricular cysts, subependymal pseudocysts, germinolytic cysts, white matter abnormalities, cortical atrophy, migration disorders, cerebellar hypoplasia, lenticulostriate vasculopathy
* *	**Features found on hearing test**
	◦ SNHL uni- or bilateral
* *	**Features found on visual examination**
	◦ Chorioretinitis, retinal hemorrhage, optic atrophy, strabismus, cataracts

GA, gestational age; ALT, alanine aminotransferase; AST, aspartate aminotransferase; SD, standard deviation.

^a^ Association between congenital CMV infection and preterm birth is not confirmed (22).

Adapted from Jones et al. (5).

The diagnosis of NPI is based on a positive CMV PCR in blood/urine or saliva in a woman known to be seropositive before pregnancy. In this group of known seropositive pregnant women, serology is not useful and has the potential to provide misleading results (4).

If there is uncertainty regarding CMV infection in children, particularly when it is not clearly definable as congenital or perinatal/postnatal ones, a retrospective evaluation of maternal serum can be conducted if blood samples from pregnant women have been preserved. However, this is possible in very limited settings.

## 6. Pathophysiology of fetal infection

Brain injury induced by CMV congenital infection may be the result of uncontrolled viral replication, immune-mediated damage by cytotoxic CD8+ T-lymphocytes, and, in the presence of placental insufficiency, fetal hypoxia (24). CMV infection of the fetus may alter the “normal blueprint” of the developing brain, affecting predominantly neural stem cells which are in abundance in the fetal brain and have an increased susceptibility to viral infection (25). Because these cells differentiate into both neurons and glia, the impact of their death or damage will result in both loss of brain mass and abnormal neuronal migration, leading to abnormal organization and communication between brain areas (25). Molecular mechanisms resulting in impaired differentiation and proliferation of neuronal stem cells are under evaluation (26–29).

It is unclear whether late-onset SNHL is caused by viral replication or by the immunological host response. Lesions in the inner ear, particularly cochlear, in fetuses are diffuse, consisting of both cytomegalic cells containing inclusion bodies and inflammation (30, 31). Vestibular and cochlear infections are frequent, and sensory structures are further altered by dysregulation in the potassium and ion circulation (30). The importance of the host immune response may be of greater importance than the viral destruction in CMV labyrinthitis, as shown in animal studies (32). This implies the potential use of an immunosuppressive agent as a therapeutic adjuvant.

## 7. Diagnosis of fetal infection

CMV DNA detected by PCR in a sample of amniotic fluid is the gold standard for the diagnosis of fetal infection. Prenatal diagnosis is performed when MPI is revealed by maternal symptoms or following prenatal serology screening or when the prenatal ultrasound is suggestive of fetal infection (4).

Amniocentesis can be scheduled after 17 weeks of gestation and at least 6–8 weeks after the suspected maternal infection (33). A negative amniocentesis preformed with a correct timing does not completely rule out the possibility of a congenital infection. The occurrence of false negatives has been reported to be as high as 8% in a recent meta-analysis (34). In fact, vertical transmission can be delayed at the placental level, resulting in a subsequent occurrence of fetal infection, and the viral load in the amniotic fluid can be insufficient to be detected using PCR analysis. However, transmission that occurs later in pregnancy is not associated with clinically relevant consequences on the children's life (34). A similar finding was described for negative chorionic villus sampling in the first trimester (35).

Prenatal ultrasound findings can be detected as late as 12 weeks after the maternal infection, so serial fetal ultrasound scans are suggested (36). Presenting features at ultrasound scan can be gross or subtle and difficult to identify. Ultrasound features can be labeled as extracerebral and cerebral findings, respectively (36) (see Table 2). Brain lesions develop only following maternal infection in the first trimester of pregnancy (35, 37). The detection of abnormal neuronal migration is challenging for prenatal ultrasound, while it can be revealed by MRI performed during the second trimester (38). The utilization of MRI at 32 weeks gestation along with serial ultrasound assessment is found to enhance the prognostic evaluation of fetal CMV infection during the first trimester. This combined approach has a high negative predictive value for both the presence of symptoms at birth and the development of moderate to severe sequelae, i.e., SNHL and/or neurological impairment (38, 39).

## 8. Diagnosis of neonatal infection

A confirmed diagnosis of cCMV is based on positive CMV DNA PCR in urine or saliva collected within 3 weeks of life (20). Although both fluids show the same sensitivity, saliva may be falsely positive because a low amount of CMV DNA from breast milk may contaminate the saliva samples. For this reason, it is recommended that the sampling process be conducted an hour after breastfeeding, and any positive results should always be verified with a subsequent urine sample. However, false positive cases showed a lower viral load in saliva, so they were easily discernible from true positive cases, which showed very high viral loads (40).

A positive CMV DNA PCR collected after 21 days of age may reflect postnatal acquisition of infection, leading to a completely different natural history and sequelae. In these cases, CMV DNA can be evaluated in the newborn dried blood spot in order to differentiate congenital to postnatal infection with a retrospective diagnosis. This test has a sensitivity of 85.7% (95% CI 74.3–92.6) (41) and helps to provide families with an explanation for clinical features clearly presenting after the age of 3 weeks. However, a delayed diagnosis means missed opportunities for improving outcomes in those children eligible for antiviral treatment. For this reason, the first step involves detecting the presence of congenital infection and searching for CMV DNA. Congenital infection has to be ruled out in fetus with IUGR not differently explained, when suggestive neonatal symptoms or signs are detected including microcephaly particularly when isolated, and in neonates failing universal hearing screening (suspicion of sensorineural hearing loss). However, most cases (95%–99%) of cCMV at birth remain undiagnosed in the absence of antenatal and/or neonatal screening programs, leading to missed opportunities (42, 43).

As such, cCMV newborn screening (NBS) programs have been developed in some states in the United States (Minnesota) (44–46) and in some Canadian provinces (Ontario by 2019 and Saskatchewan by 2022).

Hearing-targeted newborn programs are the most common (as opposed to universal screening programs) and limit cCMV screening to infants who fail their newborn hearing screening (44, 47). This approach ensures that a diagnosis is made within a suitable timeframe to enable antiviral treatment to be initiated, has proven to be cost-effective (46), and has shown high acceptance among parents (48). Targeted screening in children who failed their hearing screening test will not detect all children eligible for treatment (49).

Baseline screening to differentiate between congenital and postnatal CMV infection is helpful for extremely premature infants (<28 weeks gestational age) who are at increased risk of symptomatic postnatal infection (21).

**Figure 1:**
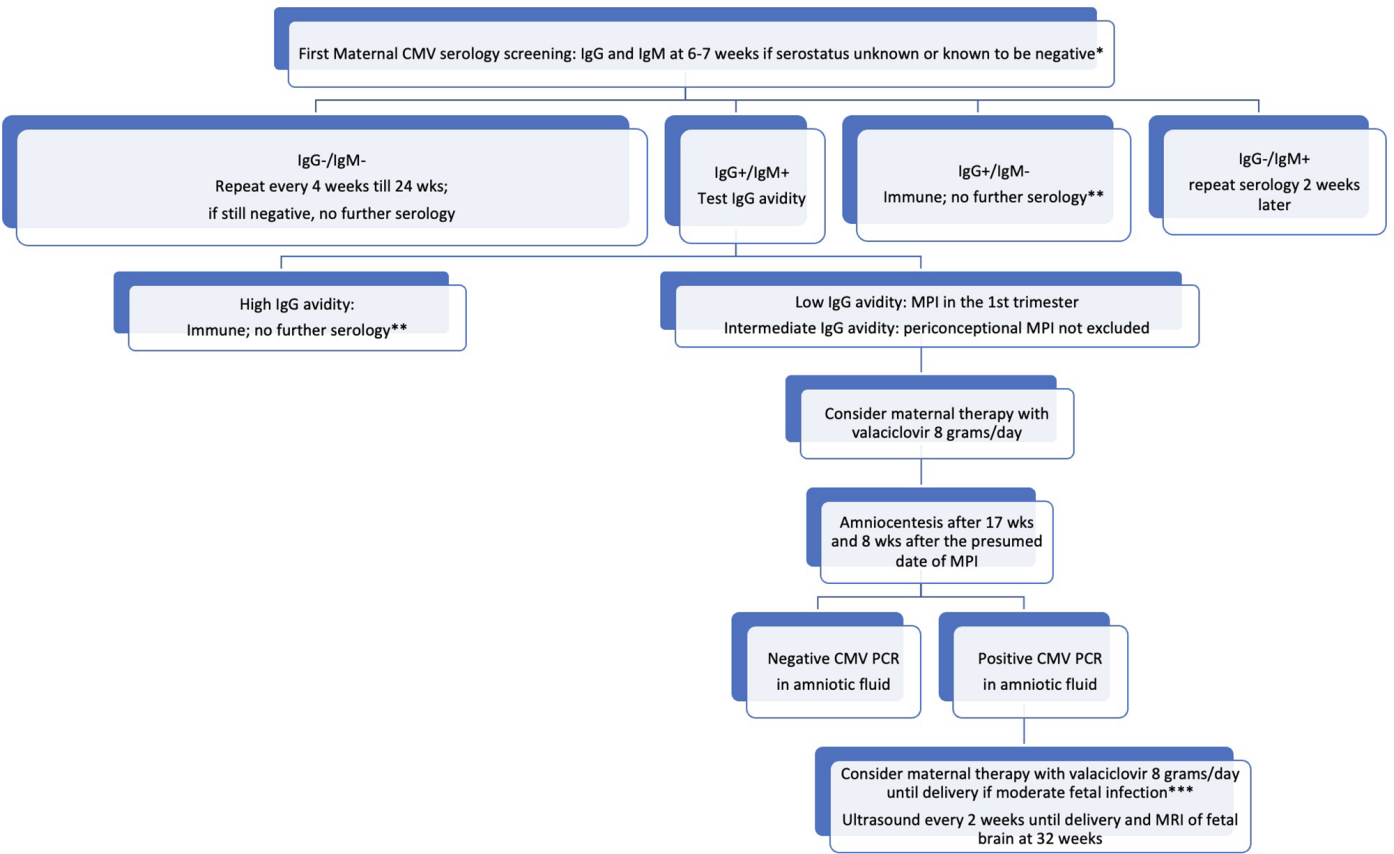
Proposed algorithm for the management of CMV infection in pregnancy. *all women regardless their serological status have to receive information about how to avoid a CMV infection. **unless maternal infection is suspected because of symptoms and/or laboratory signs and/or a fetal infection is suspected. ***signs of moderate fetal infection are listed by Leruez-Ville et al. (108); in cases of severe fetal infection, termination of pregnancy may be discussed with the parents.

In Figure 1, we propose an algorithm modifying the one by Leruez-Ville and Ville (50). In contrast to the proposal presented by the French group, our suggestion is to conduct repeated serology evaluations up to 24 weeks of gestation in seronegative women. This recommendation is based on the availability of valaciclovir in certain countries, such as Italy, up until that gestational age. Considering that antiviral therapy is currently proposed for MPI, we do not suggest repeating serological evaluation in seropositive women, unless there is a suspicion of a maternal and/or fetal infection.

## 9. Neonatal clinical features

In neonatal age, cCMV infection can present without clinically detectable features and historically defined as asymptomatic. Features usually associated with cCMV infection are reported in Table 2. The definition of asymptomatic or symptomatic status at birth changed over time, since it was based not only on clinically detectable features, but also on laboratory and instrumental evaluations. For example, SNHL was not included in some definitions (51) so that neonates with isolated SNHL were classified as “asymptomatic” in some manuscripts. The most recent definitions include the results of audiological evaluation (20, 21), but are not perfectly overlapping (Table 3).

**Table 3:** Classification of symptomatic patients (20, 21).

	Luck et al.	Rawlinson et al.
Severe	▪ Central nervous system (CNS) involvement (abnormal neurologic or ophthalmologic examination, microcephaly or neuroimaging consistent with cCMV disease [such as calcifications, moderate to severe ventriculomegaly, cysts, white matter changes, cerebral or cerebellar hypoplasia, hippocampal dysplasia, neuronal migration abnormalities]) or with life-threatening disease	▪ Central nervous system involvement such as microcephaly, radiographic abnormalities consistent with cytomegalovirus central nervous system disease (ventriculomegaly, intracerebral calcifications, periventricular echogenicity, cortical or cerebellar malformations), abnormal cerebrospinal fluid indices for age, chorioretinitis, sensorineural hearing loss, or the detection of cytomegalovirus DNA in cerebrospinal fluid, or
	▪ Eidence of severe single-organ disease (including those with clinically significant liver enzyme abnormalities [liver “failure”] and marked hepatosplenomegaly) or those with significant multiorgan involvement	▪ Multiple manifestations attributable to congenital cytomegalovirus infection: thrombocytopenia, petechiae, hepatomegaly, splenomegaly, intrauterine growth restriction, hepatitis (raised transaminases or bilirubin)
	▪ Babies with transient or otherwise clinically insignificant abnormalities (i.e., the babies are not “sick”) that resolve spontaneously over a few weeks are not included in this group even if these abnormalities are multiple	
Moderate	This group is heterogeneous and includes, for example, those with persistent (i.e., more than 2 weeks duration) abnormalities of hematologic/biochemical indices or more than two “mild” disease manifestations (as listed earlier).	(Severe and moderate patients are classified together)
Mild	Isolated (1 or 2 at most) clinically insignificant or transient findings, such as petechiae, mild hepatomegaly or splenomegaly or biochemical/hematologic abnormalities (such as thrombocytopenia, anemia, leukopenia, borderline raised liver enzyme abnormalities or conjugated hyperbilirubinemia) or SGA (defined as weight for gestational age <−2 SD) without microcephaly	One or two isolated manifestations of congenital cytomegalovirus infection that are mild and transient (i.e., mild hepatomegaly or a single measurement of low platelet count or raised levels of alanine aminotransferase)
Isolated SNHL	Not described	Sensorineural hearing loss (≥21 dB)
Asymptomatic	No signs and symptoms related to cCMV	No apparent abnormalities to suggest cCMV disease, and normal hearing

To assess features of infected newborns, all should be evaluated with a clinical examination that includes growth parameters, diagnostic auditory brain stem responses, ophthalmological examination for retinitis or retinal scarring, full blood count to assess bone marrow function, and renal and liver function tests (20).

Regarding the evaluation of the central nervous system (CNS), cranial ultrasound (CUS) and brain MRI are considered complementary tests by Rawlinson et al. (20), while MRI should be performed in babies with clinically apparent disease at birth, with clinically detectable neurologic findings including SNHL and chorioretinitis or if CUS showed abnormalities, according to European experts (21). MRI could be offered in other high-risk patients (e.g., first trimester infection).

Abnormal postnatal neuroimaging is reported in 70% of symptomatic infants (52). MRI is most sensitive to detect neuronal migration disorders (that are expression of fetal infection before 18–20 weeks of gestation and suggestive of a worse neurological outcome), cysts, ventricular dilatation or volume loss, and abnormalities of white matter signal (53). CUS is more sensitive than MRI in detecting calcification without the risk of radiologic exposure that was prevalent in the use of computed tomography in previous years. However, although CUS is the safest neuroimaging technique, it performs less well in detecting some brain abnormalities that can be associated with a poor neurodevelopmental outcome (54). The ability of neonatal neuroimaging to predict neurodevelopmental outcomes at 5–6 years of age remains uncertain, although it appears to be high (54, 55).

One-tenth of infected neonates presents with signs, laboratory abnormalities, or intracranial abnormalities in the newborn period (56), accounting for 8%–21% of all instances of congenital SNHL at birth (57). The remaining 90% of infants presented with asymptomatic infection in the neonatal period. This group was described as at risk for audiological and vestibular sequelae (58, 59). However, this concern seemed to be reduced according to more recent data (60), and it needs to be evaluated according to the more recent definition of birth status.

The disease's spectrum is similar after primary and non-primary infection regarding neonatal features as well as long-term sequelae (13, 61). This confirms that pre-existing maternal immunity provides only limited protection to the fetus.

SNHL is more common when abnormalities on a neonatal brain US and/or MRI are detected, with sensitivity and specificity around 52.9% and 90%, respectively (62).

Blood viral load is higher in children with symptoms at birth, and children with undetectable or low blood viral load (<1,000 UI/ml) seems to have a better long-term prognosis (63–66). There are different published thresholds for patients who are at higher risk of long-term sequelae, and positive predictive value varies in different series (63–66).

## 10. Long-term sequelae in infected children

The majority of children with cCMV do not develop any consequences related to the infection. However, moderate to severe long-term impairment is diagnosed in almost 25% of infected children considered as a whole (67). This prevalence is even higher among those who are symptomatic at birth (50%–70%) (51, 68), frequently presenting as hearing loss (69), neurological disability, i.e., cerebral palsy (70) (up to 10% of all cases are due to CMV), and delayed psychomotor development (71, 72). An overall mortality rate was reported as 0.5% (51).The main predictor of long-term sequelae is the gestational age at maternal infection. The relationship between gestational age and outcome of cCMV was not well defined until recently when serological timing of MPI became more accurate. In recent years, long-term sequelae were solely seen in children infected after MPI in the first trimester (<14 weeks of gestational age), while no long-term sequelae were reported in those infected after the primary infection in the second or third trimester (37). In fact, at a median follow-up of 24 months, the proportion of sensorineural hearing loss and/or neurologic sequelae was 32.4% after a maternal primary infection in the first trimester (37). These results suggest that a CMV infection can be severe only when the virus hits the fetus in the embryonic or early fetal period. When SNHL and/or neurologic symptoms are found in children with an infection occurred later in pregnancy, etiologies other than CMV should be investigated (37, 62). The risk of long-term sequelae according to the time of maternal NPI remains uncertain, but it is hypothesized that it is comparable to that observed in cases of primary infection (4).

cCMV impacts the health-related quality of life of the individual and of the family, even in cases of less severe illness (73, 74). cCMV is also a public health concern due to the care, corrective measures, and rehabilitation needed by survivors with sequelae. In fact, there are direct healthcare costs, but also indirect costs, such as caregiver burden and quality of life (75). The annual health cost attributed to cCMV infection was estimated to be $1.86 billion and £732 million in the United States and in the United Kingdom, respectively (76, 77). cCMV infection causes far more long-term sequelae than either *Haemophilus influenzae b* or congenital rubella infections prior to the availability of vaccines for these infections. The incidence of children with disabilities in the United States resulting from cCMV infection surpasses that of trisomy 21, fetal alcohol syndrome, or spina bifida on an annual basis (78).

### 10.1. Hearing loss

CMV damages the whole cochlea, affecting equally high and low frequencies (base and apex of the cochlea, respectively).

The possibility of late-onset hearing loss, progression, improvement, and fluctuation of hearing threshold make it very difficult to predict outcome and standardize follow-up of infected children. The risk for developing SNHL was reported between 12% and 20% (62, 79, 80), with certain studies even reporting rates as high as 65% (69).

Follow-up is recommended up to the age of 4–6 years (20) although late-onset SNHL most often develops within the first 3 years of life (62). Recent evidence underlined that cCMV-infected infants who do not exhibit clinical symptoms and SNHL within the first month of life have a significantly low risk of delayed hearing impairment. Therefore, it is recommended to develop a personalized follow-up plan for each patient based on their individual risk factors (60).

Middle-ear problems are frequently observed in young children and can influence in a significant way the evaluation of hearing thresholds. For this reason, it is necessary to conduct a thorough examination of the ear, nose, and throat, which should include the use of (high-frequency) tympanometry.

The need of some kind of hearing amplification was described in approximately 30% of the infected children (69), while it was lower (5.7%) in a more recent cohort (62). Cochlear implantation improves audition and language in cases with severe to profound hearing loss (81, 82).

### 10.2. Vestibular problems

Vestibular and balance dysfunctions have been reported in children with congenital CMV infection, both in those with and without hearing loss (83). All cases evaluated regarding the time of maternal infection followed the primary infection during the first trimester (37).

Although available data are of low/moderate quality, vestibular assessment should be performed as part of a neurodevelopmental follow-up in children with cCMV (83). This approach enables the early identification of children who might benefit from appropriate rehabilitation to ensure normal balance and motor development. Case–controlled longitudinal studies are required to more precisely characterize vestibular dysfunction and differentiate it from neurological impairment in order to carry out specific early supportive interventions.

### 10.3. Neurological sequelae

cCMV infection can cause developmental delay, cognitive impairment, neuromuscular dysfunction, such as cerebral palsy, epilepsy, and impaired vision function. In addition, it has been associated with autism spectrum disorder. Children with symptomatic infection at birth have a 40%–70% risk of neurological sequelae (51, 84), while those classified as asymptomatic at birth are not at increased risk of intellectual disability or motor deficits (58).

Microcephaly was the most specific predictor of intellectual disability (100%; 95% CI 84.5–100) and major motor disability (92.3%; 95% CI 74.8–99) (85).

An abnormality detected by a CT scan was the most sensitive predictor for intellectual disability (100%; 95% CI 82.3–100) and motor disability (100%; 95% CI 78.2–100). An abnormal cranial CT scan indicates a 5.6–24 times higher risk for severe sequelae and also shows a good negative predictive value (85). MRI is better than cranial US for detecting white matter abnormalities, polymicrogyria, lissencephaly, hippocampal dysplasia, and cerebellar hypoplasia (54, 86).

Epilepsy occurs in approximately 10% of patients with symptomatic infection and is often associated with other evidence of CNS damage such as migration disorders and ventricular dilatation (87). An association of cCMV infection with autism spectral disorder (ASD) has been suggested since 1980s. Despite the observed association, its role as a risk factor for ASD remains to be defined, and there is an urgent need for further studies to clarify this issue (88). A recent study conducted in Netherlands revealed a higher incidence of language development issues, concentration difficulties, and diminished quality of life compared with a control group at school age (89).

### 10.4. Ocular and visual abnormalities

Ocular abnormalities due to cCMV infection, mainly chorioretinitis, often presenting as retinal scarring, have been recognized almost exclusively in patients who were symptomatic at birth, although the prevalence of ocular abnormalities has varied widely from no cases to a rate as high as 40% (90–92). In children without ocular abnormalities at birth, there is no evidence of the benefits of a prolonged visual follow-up, since the postnatal development of chorioretinitis is very unlikely (90).

### 10.5. Olfactory function

CMV infection leads to both olfactory and hearing impairments in a mouse model. However, little is known regarding olfactory dysfunction in CMV-infected children, partly because it is challenging to assess olfaction in toddlers. A recent study on a small cohort of patients (34) concluded that cCMV infection is associated with reduced olfactory performance in children with infection symptoms at birth (93). This new field appears to possess relevant clinical implications as the loss of olfactory function can impact nutrition, social interaction, safety, and overall quality of life. Detecting olfactory disorders at an early stage may facilitate the implementation of olfactory rehabilitation programs in order to limit neurodevelopmental consequences.

## 11. Prevention and treatment of congenital CMV infection

Damage due to cCMV infection may be prevented at various levels including maternal immunization, maternal awareness to prevent infection in pregnancy, prenatal diagnosis of congenital infection followed by antiviral treatment, and neonatal screening to identify the infected babies who could receive antiviral agents when indicated in order to prevent sequelae or reducing the damage and could be included in a monitoring program for the early detection and correction of sequelae.

### 11.1. Prevention of maternal infection

Toddlers show prolonged viral shedding for weeks or even months (94) and are a significant source of infection in women and therefore a risk for cCMV infection in their offspring (95).

Hygienic measures aim to avoid direct contact with the saliva and urine of young children that are the most common sources of infection in pregnant women (96). They include handwashing after exposure to young children's body fluids as well as surfaces touched by children (toys, high chair, stroller, etc.) and avoiding kissing children on the mouth/cheeks and sharing utensils, food, drinks, washcloths, etc. An intervention based on the identification and hygiene counseling of CMV-seronegative pregnant women significantly prevents maternal infection (1.2% rate of seroconversion in the intervention group with hygienic counseling at 11–12 weeks of gestation vs. 7.6% in the control group) (97). However, in order to prevent potential severe cases related to periconceptional infections during the first trimester of pregnancy, increasing awareness before pregnancy should be the target of information campaigns. Moreover, previous data suggest that the prevalence of cCMV decreased dramatically in 2020 compared with 2019, coinciding with the COVID-19 pandemic (98), confirming that hygiene intervention is effective in reducing CMV infection in pregnancy. Despite the impact that cCMV has on newborn health, awareness among healthcare providers (99) and also in the general population is suboptimal, as recently demonstrated among Italian people (100). In order to improve awareness, a specific program should be offered to all female population, not only to seronegative women, considering the risk of transmission and symptoms after NPI. All available studies on awareness have been conducted on the general population or have specifically focused on the female group, which is the main target for prevention efforts. However, it is important to ensure that fathers are correctly informed as well, as CMV has the potential to spread across the whole family, particularly when mothers exhibit immunosuppression, such as in cases of HIV infection, which is a known risk factor for the transmission of cCMV to the offspring (101).

An efficacious prevention of congenital infection requires a vaccine that can effectively protect the mother against both primary and non-primary infections. A vaccine administered to 12-year-old girls with the goal of preventing maternal CMV infections during pregnancy was defined as a top national priority in the United States. Encouraging data are emerging from clinical trials, but a human CMV vaccine has not been licensed yet (102). Both human CMV live vaccines (e.g., live-attenuated, chimeric, viral-based) and non-living vaccines (subunit, RNA-based, virus-like particles, plasmid-based DNA) have been investigated. The major difficulties in developing a satisfactory vaccine include human CMV's capacity to evade the immune response, unclear immune correlates for protection, low number of available animal models, and insufficient general awareness. Moreover, there is a need to determine the best target populations for vaccine administration.

### 11.2. Antenatal treatment to prevent and/or treat fetal infection

Several strategies of prenatal treatment to reduce the risk of vertical CMV transmission when the mother is infected have been studied. Hyperimmune globulins (HIGs) have shown contradictory results in different studies (12, 103–106). In an uncontrolled study published in 2005, the administration of CMV-specific hyperimmune globulin at a dose of 100 U/kg intravenously monthly to pregnant women with primary CMV infection significantly reduced the rate of intrauterine transmission from 40% to 16% (105). These results were not confirmed by a double-blind randomized, placebo-controlled trial where the transmission rate was not significantly lower in treated women (30% vs. 44% in placebo) and the clinical outcome of congenital infection at birth was similar in the two groups. In addition, the number of obstetrical adverse events such as premature delivery was higher in the hyperimmune globulin group than in the placebo group (13% vs. 2%) (12). Another placebo-controlled randomized controlled trial was stopped for futility at interim analysis after evaluating results on half of the foreseen cases because the transmission rate was 22.7% vs. 19.4%, with similar rates of preterm birth (104). However, a recent non-randomized phase I study (106) reported that a biweekly administration of a 200 U HIGs showed a marked decrease in the risk of maternal–fetal transmission compared with a historical cohort (7.5% vs. 35%) in the context of a systematic serology screening in pregnancy. HIGs were administrated in a very selected population, in an early stage of pregnancy with a recent primary infection, so this data should be in interpreted with caution (106). Within this context, HIG seems to be safe.

In recent years, antiviral treatment during pregnancy has arisen as a novel prophylactic treatment after a maternal primary infection. In a double-blind, placebo-controlled RCT, the administration of valaciclovir (8 g/day) started soon after the confirmation of MPI in early pregnancy (periconceptional period and the first trimester) and continued until the occurrence of diagnostic amniocentesis, which reduced the rate of vertical transmission by 71% (11.1% vs. 29.8% in the placebo group with an odd ratio of 0.29) (107). Moreover, a phase II multicenter open-label study showed that the same high-dose of valaciclovir (8 g/day) from the diagnosis of fetal infection in the second trimester to delivery in women carrying a CMV-moderately infected fetus was associated with a higher proportion of asymptomatic infected neonates (82% vs. 43%) (108). No maternal, fetal, or neonatal adverse effects were reported. A recent real-life observational study conducted in multiple centers in Italy confirmed that valaciclovir significantly reduces the cCMV rate at the time of amniocentesis with a good tolerability profile and showed that the treatment was associated with reduction of termination of pregnancy and symptomatic cCMV at birth (109).

### 11.3. Neonatal therapy

Ganciclovir (GCV) or its prodrug valganciclovir (VGCV) is the preferred antiviral agent for treating congenital CMV disease (and not only infection). In fact, at present, antiviral therapy with (val)ganciclovir is restricted to neonates with symptomatic congenital CMV disease involving the central nervous system (20, 21). Due to lack of evidence, full consensus could not be reached on how to approach children with moderate disease, and treatment decisions are currently made on a case-by-case basis (20, 21).

A course of 6 weeks of intravenous GCV improved the hearing and neurodevelopmental outcomes in infants with symptomatic cCMV involving the CNS (110, 111). Currently, antiviral therapy with oral VGCV for 6 months is the standard of care for infants with symptomatic disease, unless the oral route is not usable. In fact, it is preferred over intravenous GCV due to its decreased incidence of significant neutropenia but appeared to improve hearing and developmental outcomes modestly in the longer term (112). It is administered at a dose of 16 mg/kg twice daily for 6 months starting in the first month of life. Therefore, antiviral therapy is recommended in cases of CNS disease (microcephaly, CNS calcification, chorioretinitis, white matter changes or other abnormalities on MRI consistent with CMV disease, other “severe” disease including life-threatening, or severe single-organ or multiorgan non-CNS disease). However, in case of “moderate” diseases (i.e., multiple minor findings consistent with CMV disease), treatment has to be considered after discussion with a specialist, and it is not recommended in case of “mild” disease that is isolated or transient diseases (e.g., jaundice, petechiae, SGA in isolation, maximum of two abnormalities) and no clinical or biochemical findings of disease (±detectable CMV viremia) (110, 111).

In all the studies (110–112), the participants were over 32 weeks of gestation at birth and started treatment before 4 weeks of age. These studies are summarized in Table 4. At the present time, there are no licensed antiviral therapies for cCMV, necessitating clinicians to rely on the interpretation of inclusion criteria and the current recommendation (20) in order to decide whether to start treatment.

**Table 4:** Randomized controlled trials of antiviral therapy for congenital CMV.

Study and setting	Inclusion and exclusion criteria	Number included	Intervention	Outcome
Kimberlin 2003 1991–1999, multicenter (110)	Inclusion: Neonates (<1 month of age) with symptomatic congenital CMV involving CNS Exclusion: <1,200 g birth weight <32 weeks of gestation at birth	100 enrolled 42 completed follow-­up (25 treatment, 17 no treatment)	Intravenous ganciclovir (6 mg/kg BD) 6 weeks vs. no treatment	-Improved or maintained normal hearing at 6 months: *n* = 21/25 (84%) treatment group vs. *n* = 10/17 (59%) control group (*p* = 0.06) - Worsened hearing at 6 months compared with baseline: *n* = 0 (0%) treatment group vs. *n* = 7/17 (41%) control group (*p* < 0.01)
Oliver 2009 1991–1999, multicenter (111)	As Kimberlin 2003	60 completed all assessments (29 treatment, 31 no treatment)	As Kimberlin 2003	- Average number of delayed milestones at 12 months: 10.06 in treatment group vs. 17.14 in control group (*p* = 0.007)
Kimberlin 2015 2008–2011, multicenter (112)	Inclusion: Neonates (<30 days of age) with symptomatic congenital CMV, including: hematological, organomegaly, intrauterine growth restriction, hepatitis, central nervous system involvement, sensorineural hearing loss Exclusion: <1,800 g birth weight, <32 weeks of gestation	109 enrolled, 96 randomized 68 completed 24 months of follow-up (37 in 6-month group and 31 in 6-week group)	Oral valganciclovir (16 mg/kg BD) 6 months vs. 6 weeks (followed by 4.5 months of placebo)	-No significant difference in “best ear” hearing at 6 months -“Total ear” hearing remained normal or improved: 73% in the 6-­month group vs. 57% in 6-week group (*p* = 0.01) at 12 months and was maintained at 24 months 77% vs. 64%, *p* = 0.04 -Higher Bayley-­III language-­composite scores at 24 months in 6 months vs. 6 weeks (*p* = 0.005) -Higher receptive-­communication scale scores at 24 months in 6 months vs. 6 weeks (*p* = 0.003)
NCT01649869 2015–2019^a^	Inclusion: Children aged 1 month to 4 years with congenital CMV and sensorineural hearing loss Exclusion: Profound sensorineural hearing loss, previous ganciclovir or valganciclovir	Target 54 32 children completed	Oral valganciclovir 16 mg/kg BD 6 weeks vs. placebo	-Awaiting formal publication -At 6 months, no difference in hearing change (neither improvement, nor deterioration)
NCT02005822 (CONCERT Trial) (113) 2012–2016	Inclusion: Children ≤13 weeks, born at term (≥37 weeks) with sensorineural hearing loss (≥21 dB) and prospectively diagnosed with cCMV through dried blood spot testing, without prior clinical suspicion Exclusion: Parental refusal	37 enrolled (25 treatment, 12 no treatment)	Oral valganciclovir 16 mg/kg BD 6 weeks vs. placebo	-Awaiting formal publication -Further deterioration of hearing at age 18–22 months is prevented

BD, twice daily; CMV, cytomegalovirus; CNS, central nervous system.

^a^ Available at: https://clinicaltrials.gov/ct2/show/study/NCT01649869.

Modified from Jones et al. (5).

There is no current consensus on treating infants with congenital cCMV and isolated SNHL (20, 21). In fact, limited high-quality evidence of efficacy is available because only few infants with this characteristic were studied prospectively (112) and beneficial data were derived from retrospective studies (114). However, a prospective nationwide non-randomized controlled trial, named CONCERT (Congenital Cytomegalovirus Infection in infants with Isolated Hearing Loss) (ClinicalTrials.gov Identifier: NCT02005822), showed that 6 weeks of VGC in infants with cCMV and hearing loss prevented further deterioration of hearing at age 18–22 months (113).

Antiviral therapy is not currently administered to infants who are diagnosed with symptomatic infection later in life. Two small retrospective observational series have reported improved hearing outcome in treating older infants (115, 116), and a small multicenter, single-arm, open-label study observed no differences in hearing efficacy between the younger (14–28 days old) and older age (31–66 days old) groups (117). However, the preliminary data of a phase II, double-blind, randomized placebo-controlled trial of children from 1 month to 4 years of age (ClinicalTrials.gov Identifier: NCT01649869) on 35 children enrolled (median age of 18.7 months) showed no impact of treatment on hearing outcomes. The data from the prospective CONCERT trial (ClinicalTrials.gov Identifier: NCT02005822) showed that 6 weeks of VGC in infants with cCMV and hearing loss prevented further deterioration if initiated within the first 3 months (113).

In conclusion, the benefits of antiviral therapy are demonstrated on short- and medium-term outcomes, particularly auditory function. Nevertheless, the benefits on development if CNS is involved are unknown.

## 12. Breast milk and CMV infection in preterm infants

Breast milk (BM) is the best source of nutrition for newborns, especially if premature. Unfortunately, there is evidence of symptomatic postnatal CMV infection acquired through maternal milk in preterm neonates (118). It should be considered in very low birth weight infants who are breastfed by seropositive mothers and presenting severe or sepsis-like symptoms with negative cultures (119).

To prevent the vulnerable tiny preterm infants from breast milk-acquired CMV infection, only heat inactivation eliminates virus infectivity. Short-term heat inactivation for 5 s at 62°C maintains the benefits of feeding breast milk without the disadvantages of CMV transmission (119).

## 13. Future perspectives

### 13.1. Awareness and prevention with hygienic measures

Despite the prevalence of congenital CMV infection and the consequences for individuals, families, and society, awareness is low among pregnant women and healthcare professionals as well.

Despite the birth prevalence of cCMV being higher than other congenital conditions [spina bifida, trisomy 21, or congenital toxoplasmosis infection (78)], CMV is less well known among women of childbearing age. Reduction of pregnant women's contact with the infected urine or saliva from young children has therefore been identified as one of the most important potential preventative strategies to reduce antenatal CMV infection (120–122). Such advice is not routinely provided as part of routine antenatal care in the majority of settings worldwide; however, advice is available online if women seek it, for example, from the Centre for Disease Control and Prevention, CMV action, and the National Health Service (NHS). Pregnant women and healthcare providers strongly agree that CMV risk reduction measures should be included in antenatal care (123).

Increasing awareness before pregnancy should be the best aim of information campaigns. While waiting for CMV vaccine to become available, they may represent a responsible and acceptable primary prevention strategy to reduce congenital CMV.

### 13.2. Maternal screening and therapy in pregnancy

Despite the heavy burden of cCMV, screening for maternal infection in pregnancy has not been recommended by any Public Health body so far (20, 124, 125). The last Italian guidelines for pregnancy (126) did not include serological evaluation for CMV.

This was due to concerns over the absence of consolidated data on epidemiology, the limited sensitivity and specificity of serologic assays available for diagnosis of maternal infection, the difficulty in establishing the prognosis of an infected fetus, and the absence of validated treatment options (50). However, enough progress has been made in recent years to fill those gaps, and nowadays CMV serology screening in the first trimester of pregnancy meets the WHO's criteria for a screening program. It is time to change Public Health policies toward systematic (universal) serology screening in pregnancy in some countries, particularly in Italy where the specific antiviral therapy is authorized since 2020 (127).

Currently, there is a demonstrated effective treatment, i.e., valaciclovir, in order to prevent the mother-to-fetus transmission of CMV and to treat infected fetuses early enough to avoid developing irreversible CNS injury (107–109). This drug was introduced free in Italy until December 2020 (127) with dual purposes of preventing vertical transmission and reducing symptoms. However, the absence of antenatal screening to identify primary maternal infection in the first trimester of pregnancy limits its potential benefits. Therefore, screening recommendations need to be kept under review and avoid any more delays.

Albeit some remaining pitfalls such as the interpretation serology in some cases (low level of IgG), the tools available for the diagnosis of maternal primary infection are reliable. Maternal non-primary infection could not be diagnosed by serology, and it could be difficult to identify seropositive pregnant women at risk of fetal transmission (50). Therefore, as previously discussed by Leruez-Ville and Ville (50), at a population level in Europe, a strategy aiming to prevent mother-to-fetus transmission would only apply to 50% of all cCMV cases, those following a maternal primary infection. However, on an individual level, this strategy could be very beneficial if applied to the seronegative pregnant women because the risk cCMV and related sequelae (neurologic and/or hearing loss) following maternal infection in the first trimester were respectively 24-fold and sixfold higher than in the general pregnant population.

A serology screening is usually well accepted. In the screening study conducted by Picone et al., only 3% of women refused to be screened (128). Moreover, the majority of pregnant Canadian women involved in a recent study want to have CMV serological screening once informed regarding congenital CMV infection (129) although an approved treatment was not available at the time of the study.

The incidence of congenital toxoplasmosis, according to early cumulative published data from the New England Newborn Screening Program over a 12-year period (1988–1999), was 0.91 cases per 10,000 live births, which would have translated to the birth of approximately 365 infants with congenital toxoplasmosis in the United States each year. The incidence of congenital toxoplasmosis decreased after 1999, and over the past 9 years (2006–2014), the incidence was approximately 0.23 cases per 10,000 live births (130). According to these data, cCMV is almost 50 times more frequent than congenital toxoplasmosis, but universal screening of toxoplasmosis in pregnancy is available in many countries, and CMV screening is limited to some areas or countries.

Incorporating CMV serological screening into an established pregnancy surveillance program is a viable option for the identification of CMV infection in pregnant women and for identification of the ones with eligibility to antiviral treatment. A possible schedule could consist of a first evaluation of specific IgG and IgM at 6–7 weeks. Subsequently, the test could be repeated every 4–6 weeks if still negative (as for toxoplasmosis), until 14 weeks of gestation, because severe cCMV infection is unlikely to occur after that gestational age (37).

Although HIGs seem safe in pregnant women, there are contradictory data about efficacy in the prevention of fetal infection. Several clinical trials did not find a lower rate of fetal infection with HIG (12, 104). However, in a cohort of selected patients (close infection) in Germany, HIG-treated women showed a low rate of vertical transmission (106).

### 13.3. Neonatal screening

Best biological samples and protocols for universal neonatal screening have still to be defined and impact of universal screening programs are keenly awaited to extend policies. In fact, cCMV screening programs raise unique ethical dilemmas of both under- and over-diagnosis of cCMV as well recently explored (131). An active debate about the cost-effectiveness of neonatal cCMV screening programs, as well as the economic burden of cCMV (75), is still going on.

In the meantime, clinical features for CMV DNA evaluation should be well known to every neonatologist and pediatrician because delayed diagnosis means missed opportunities for improving outcomes in those eligible for treatment.

### 13.4. Definition of neonatal onset

The definition of asymptomatic or symptomatic status at birth changed over time because initially it was based only on clinically detectable features while over time also laboratory and instrumental evaluations were included in the evaluation. These “new” criteria are essential to estimate better the impact of long-term sequelae in the two groups of symptomatic and asymptomatic children in order to undergo a better counseling for family at the time of diagnosis and to establish a targeted follow-up plan.

Regarding the evaluation of CNS involvement, MRI may serve as a complementary technique to cranial US. MRI should be recommended in children with symptoms at birth, as well as neonates with hearing loss due to CMV infection, chorioretinitis, or abnormalities in cranial ultrasonography. There is no consensus among experts if MRI should be recommended to all children at risk of long-term sequelae (i.e., first trimester infections).

Moreover, it is not always simple to inbox the single patient in only two categories (asymptomatic vs. symptomatic), and it could be useful to study the follow-up of these “gray-zone” patients in order to better define a targeted follow-up plan.

### 13.5. Therapy in children

Currently, there are no antiviral drugs that have been officially approved for treating cCMV infection so that clinicians have to use them off-label, with all documentation needed. Prescription could become easier in a situation that is now well defined by RCTs and recommendations.

Due to a lack of evidence, full consensus on how to approach moderately symptomatic children could not be established, and treatment decisions are currently made on a case-by-case basis. Development of a validated clinical scoring system for disease severity at presentation and risk of sequelae would be beneficial for both counseling parents and informing treatment decisions.

Moreover, there is a scarcity of data to recommend the start of antiviral treatment in preterm infants and less symptomatic children. Evidence is needed before starting therapy in children with isolated SNHL and symptomatic children diagnosed beyond the neonatal period.

In addition, GCV and VGCV have been used since 2000s in newborns, but there are potential concerns with regard to long-term toxicities, such as impact on fertility (derived from animals). Recording information on infected children through international registries is important to monitor rare and long-term outcomes. One is the European CCMVNET registry.

Promising newer antivirals, such as letermovir and maribavir, have recently been approved for the prevention or treatment of CMV in the transplant setting. Studies investigating their use in congenital CMV are being planned.

The importance of the host immune response in the audiological damage, more than viral destruction, suggests that an immunosuppressive agent might be useful as a therapeutic adjuvant in addition to antiviral treatment (32). Further research is needed.

### 13.6. Follow-up in children

A CMV infection can be severe only when the virus hits the fetus in the embryonic or early fetal period. Recent guidelines recommend auditory follow-ups for at least 5 years for all infected children. This raises parental anxiety and generates significant costs. An auditory and specialized neurologic follow-up may be highly recommended in cases of a maternal infection in the first trimester (<14 weeks) (37). Children with negative amniocentesis have a good prognosis, and long-term sequelae are very unlikely (34).

## 14. Conclusions

Congenital CMV infection poses a significant burden, not only on the patient but also on his/her family and society. To date, some challenges remain, particularly the opportunity of a universal serological antenatal screening for subsequent valganciclovir treatment during pregnancy, when appropriate.
